# Impact of chronic potassium binder treatment on the clinical outcomes in patients with hyperkalemia: Results of a nationwide hospital-based cohort study

**DOI:** 10.3389/fphys.2023.1156289

**Published:** 2023-04-12

**Authors:** Eiichiro Kanda, Naru Morita, Toshitaka Yajima

**Affiliations:** ^1^ Medical Science, Kawasaki Medical School, Okayama, Japan; ^2^ Cardiovascular, Renal, and Metabolism, Medical Affairs, Osaka, Japan

**Keywords:** hyperkalemia, potassium binders, serum potassium, chronic treatment, hospital claims

## Abstract

**Introduction:** Hyperkalemia (HK) is a common disorder in patients with heart failure or chronic kidney disease, and potassium binders (PBs) are recommended to control serum potassium (S-K) levels. Although HK is often a chronic condition, short-term and intermittent PBs treatment has been largely applied to control S-K levels, and little is known about the impact of long-term and chronic PBs treatment on clinical outcomes.

**Method:** This retrospective cohort study was conducted using a Japanese claims database (April 2008–September 2018). HK was defined as at least two S-K ≥5.1 mmol/L within a 12-month(M) interval. The index date was defined as the initial PB prescription date, and the S-K values were examined at 3M, 6M, and 12M after the index. The medication possession ratio (MPR) was used to evaluate the length of the prescribed period of PB, as prescription refill was not allowed in Japan. Clinical outcomes were analyzed by comparing MPR <80% to MPR ≥80% using Cox proportional hazards regression.

**Results:** We found 4,321 patients with HK and were on initial PB treatments, and 993 and 3,328 patients were categorized in the MPR <80% and MPR ≥80% groups, respectively. The mean prescription days ±SD in the MPR <80% and MPR ≥80% groups were 114.7 ± 9.1 and 1151.2 ± 22.5, respectively. S-K value with adjustment by covariates in MPR <80% and MPR ≥80% groups were 5.62 (95% CI: 5.57–5.68) and 5.72 (95% CI: 5.68–5.76) at index followed by 4.65 (95% CI: 4.58–4.71) and 4.57 (95% CI: 4.51–4.62) at 3M, respectively. The hazard ratios of incidence rates in hospitalization was 1.41 (*p* < 0.001), introduction of renal replacement therapy was 1.25 (*p* < 0.003), recurrent HK was 1.67 (*p* < 0.001), and decreased eGFR was 1.41 (*p* < 0.001), respectively.

**Conclusion:** These results indicate a higher risk of adverse outcomes when PBs were not prescribed chronically, whereas S-K levels were similarly controlled. Chronic control with continued PBs rather than temporary treatment may be associated with the reduction of adverse clinical outcomes in patients with HK.

## 1 Introduction

Serum potassium (S-K) levels play an important role in maintaining homeostasis in humans. In cells such as neurocytes and myocytes, abnormalities in the resting membrane potential due to elevated S-K levels—hyperkalemia (HK)—can lead to the impairment of action potential transmission and muscular function and, in severe cases, can result in life-threatening conditions such as fatal arrhythmia and cardiac arrest (Gumz et al., 2015; Sarwar et al., 2016; Montford and Linas, 2017). In the chronic course of HK, it has been shown that there is a U-shaped relationship between S-K levels and mortality in patients with a worse prognosis if their potassium levels become higher or lower than the normal range (Korgaonkar et al., 2010; Nakhoul et al., 2015; Luo et al., 2016; Collins et al., 2017; Krogager et al., 2017; Furuland et al., 2018; Kashihara et al., 2019). Moreover, HK places a significant economic burden on healthcare expenditures and resource utilization (Gumz et al., 2015; Mu et al., 2020).

The management of HK varies according to its underlying cause (Kashihara et al., 2019). Recent guidelines from the European Society for Cardiology (ESC) for acute and chronic heart failure (HF) defined HK and its clinical severity as mild, moderate, and severe when S-K levels are >5.0 to <5.5 mmol/L, 5.5–6.0 mmol/L and >6.0 mmol/L, respectively (McDonagh et al., 2021). While the treatment for acute or life-threatening HK is well-defined (Clase et al., 2020), the management of chronic HK is varied, and there is no standard care or specific guidelines in Japan. In general, treatment consists of a combination of various approaches, such as a low-potassium diet (Palmer and Clegg, 2016), correction of metabolic acidosis, down-titration or discontinuation of medications associated with HK, prescription of diuretics, and the use of cation-exchange resins or binders (Clase et al., 2020) depending on the condition of each patient.

In 2022, The American Heart Association/American College of Cardiology/Heart Failure Society of America Guideline for the Management of Heart Failure (HF) recommended the use of potassium binders (PBs) in HF patients who experienced HK while taking renin-angiotensin-aldosterone system inhibitors (RAASi) (Heidenreich et al., 2022). The Kidney Disease Improving Global Outcomes (KDIGO) guidelines also recommend considering measures to reduce S-K levels, such as PBs, for the management of HK associated with the use of RAASi before implementing RAASi down-titration or discontinuation (Clase et al., 2020).

However, PBs have several characteristics and side effects that limit their use, such as poor tolerability, high incidence of constipation, and severe gastrointestinal complications, including intestinal necrosis. Poor palatability, such as a peculiar smell or taste, makes it difficult to continue taking PB medications. It has been reported that approximately one-quarter of patients receiving calcium polystyrene sulfonate (CPS) do not adhere to the treatment (Gorriz JL et al., 2019). A previous study that examined real-world evidence of Japanese patients with HK reported that PBs consisting of CPS and sodium polystyrene sulfonate (SPS) were prescribed to only 5.8% of the patients when the first S-K levels over 5.0 mmol/L were identified (Kashihara et al., 2019). In the follow-up period, the prescription rate increased to 24.0%, although 100% of the patients were prescribed lower daily doses than standard doses. Differences in PB prescription rates were observed among the countries. A retrospective survey of recurrent HK in five European countries reported that PB prescription rates in France, Spain, Italy, Germany, and the United Kingdom were 45.4%, 21.5%, 13.4%, 8.1%, and 7.5%, respectively (Rossignol et al., 2020). This suggests differences in the treatment strategies for HK from country to country, even in Europe. Heterogeneity was also observed on the days of PB prescription. A study using the provincial administrative health database of Canada reported that 22% of patients who were prescribed PBs received a 1-day supply, and approximately 65% of those received their first prescription for ≤10 days (Ren et al., 2022). Other studies from the US also reported that PB prescription rates at 30, 60, and 90 days after the initial prescription were 20%–25%, 5%, and 2%, respectively (Desai et al., 2020; Kovesdy et al., 2020). In Japan, it has been reported that the median duration of PB treatment is 140 days (Kashihara et al., 2019), which is significantly longer than in other countries. However, evidence of the clinical benefits of long-term potassium binder therapy in real-world clinical settings remains scarce (Heidenreich et al., 2022).

In the current study, we sought to assess the effects of long-term, chronic treatment with a PB on S-K control by dividing patients into two groups based on the medication possession ratio (MPR), using a threshold of 80% as an indicator of long-term and chronic use (Karve et al., 2009; Kuwabara et al., 2015; Burge et al., 2016; Baumgartner et al., 2018; Hartman et al., 2019; Higa et al., 2022; Komatsu et al., 2022). We also assessed the clinical outcomes in patients with HK to understand optimal management in real-world clinical practice.

## 2 Materials and methods

### 2.1 Data source

A Japanese hospital claims database from Medical Data Vision Co., Ltd., was used for the analysis. The database has nearly 40 million inpatient or outpatient data accumulated since April 2008 from approximately >400 hospitals, compiles administrative claims data and laboratory data in which diagnoses are coded according to the International Classification of Diseases, 10th revision (ICD-10) and treatments are recorded using Anatomical Therapeutic Chemical Classification (ATC) codes. All data were de-identified and anonymous (Tanaka et al., 2015).

### 2.2 Study design

**FIGURE 1:**
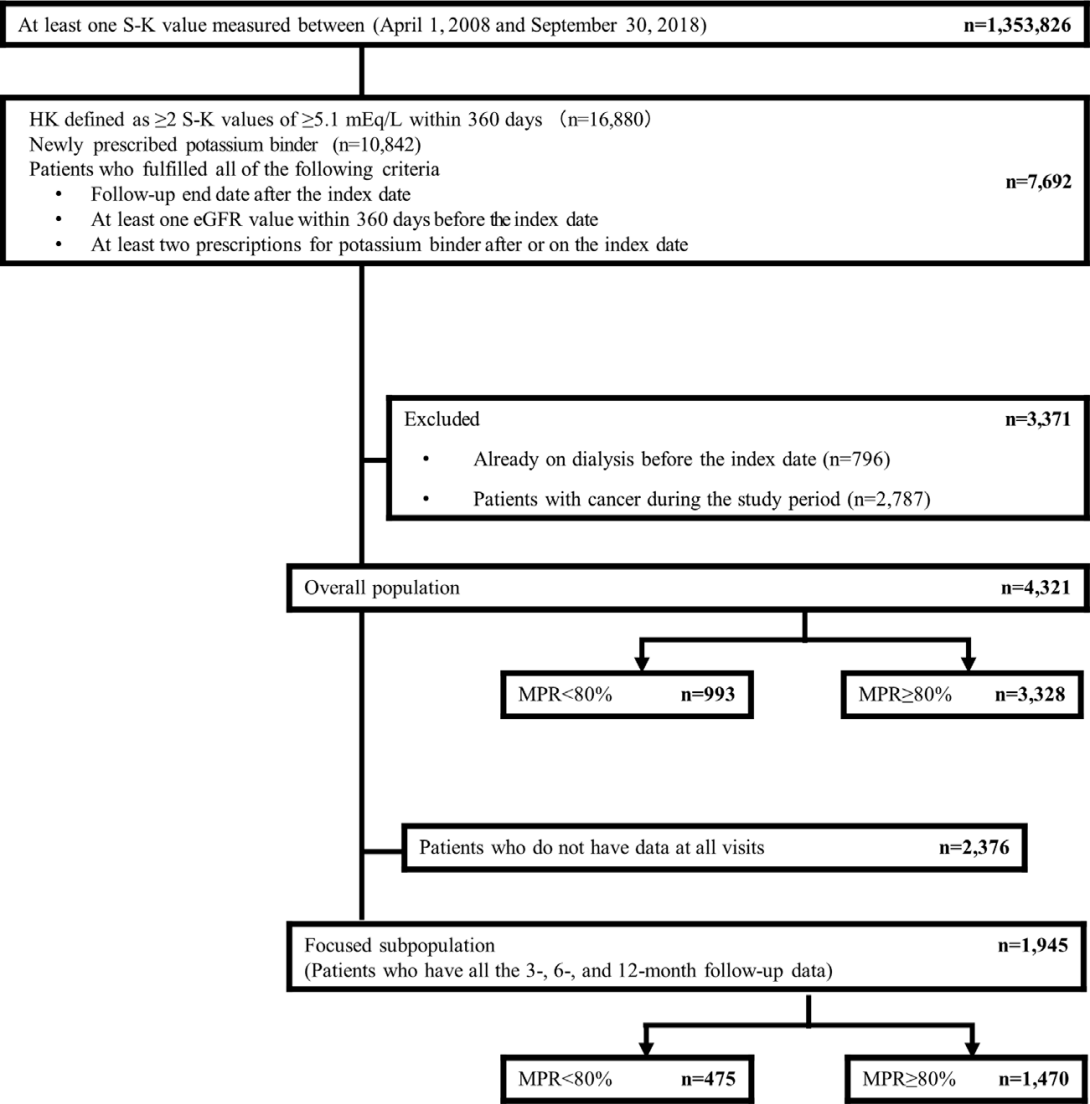
Flow diagram of patient inclusion in the study.

We first extracted 1,353,826 patients who had at least one S-K value measured during the study period (1 April 2008 to 30 September 2018) from the database. Of these patients, 16,880 met the criteria of HK, defined as ≥2 S-K values of ≥5.1 mEq/L within 360 days, and 10,842 patients were newly prescribed a PB (Figure 1). The date of PB prescription was defined as the index date, and patients with baseline data collected ≥12 months prior to the index date were eligible. Other eligibility criteria were age ≥18 years at the index date, at least one measurement of estimated glomerular filtration rate (eGFR) within 360 days before the index date, and ≥2 prescriptions for PB on or after the index date (Supplementary Figure S1). Patients were excluded if they had undergone dialysis before the index date or had a cancer diagnosis at any time during the study period. Finally, 4,321 patients met all the inclusion and exclusion criteria and were defined as the overall population, in which 1,945 patients who had all the 3-, 6-, and 12-month(M) follow-up data were defined as the focused subpopulation.

The eligible patients were then divided into two groups according to the MPR, where 993 and 3,328 patients were categorized in the MPR <80% and MPR ≥80% groups, respectively, for the overall population (Table 1), and 475 and 1,470 for the focused subpopulation (Supplementary Table S2). The MPR was used as an indicator of the duration of PB treatment since refillable prescriptions were not accepted in Japan (Akaba et al., 2019). Calculations were performed as the percentage of the sum of days of prescription for all fills in the follow-up period to the number of days in the follow-up period (Karve et al., 2009; Baumgartner et al., 2018). The patients were followed up from the index date until death, the end of registry, or drop out of the database. Key clinical events examined during the follow-up period included death, emergency room (ER) visits, hospitalization for any reason, hospitalization for HF, rehospitalization, introduction of renal replacement therapy (RRT), cardiac events, recurrence of HK, and decline in eGFR. Detailed definitions and other clinical events are presented in Supplementary Table S1. Chronic kidney disease (CKD) stage was stratified based on eGFR, according to the Japanese Society of Nephrology evidence-based clinical practice guidelines for CKD 2018 (Japanese Society of Nephrology, 2019).

**TABLE 1:** Patient characteristics at the index date (overall population).

	All patients	MPR <80%	MPR ≥80%
	(*n* = 4321)	(*n* = 993)	(*n* = 3328)
Age (years)	74.6 ± 12.83	74.6 ± 12.24	74.6 ± 13.01
Sex, male	2475 (57.3%)	544 (54.8%)	1931 (58.0%)
ADL	13.7 ± 7.80	13.7 ± 7.72	13.7 ± 7.83
eGFR (mL/min/1.73 m^2^)	28.5 ± 20.8	27.2 ± 19.2	28.9 ± 21.2
Recorded diagnoses			
Diabetes mellitus	2447 (56.6%)	559 (56.3%)	1888 (56.7%)
Hypertension	3496 (80.9%)	810 (81.6%)	2686 (80.7%)
Heart failure	1907 (44.1%)	419 (42.2%)	1488 (44.7%)
CKD	3018 (69.8%)	703 (70.8%)	2315 (69.6%)
Stage 1^a^	15 (0.5%)	3 (0.4%)	12 (0.5%)
Stage 2^a^	78 (2.6%)	11 (1.6%)	67 (2.9%)
Stage 3a	155 (5.1%)	37 (5.3%)	118 (5.1%)
Stage 3b^a^	495 (16.4%)	93 (13.2%)	402 (17.4%)
Stage 4^a^	1140 (37.8%)	291 (41.4%)	849 (36.7%)
Stage 5^a^	1135 (37.6%)	268 (38.1%)	867 (37.5%)
Myocardial infarction	213 (4.9%)	44 (4.4%)	169 (5.1%)
Peripheral vascular disease	951 (22.0%)	212 (21.3%)	739 (22.2%)
Cerebrovascular disease	1194 (27.6%)	273 (27.5%)	921 (27.7%)
Chronic pulmonary disease	828 (19.2%)	205 (20.6%)	623 (18.7%)
Atrial fibrillation/atrial flutter	677 (15.7%)	151 (15.2%)	526 (15.8%)
Obesity	22 (0.5%)	4 (0.4%)	18 (0.5%)
Acute kidney injury	341 (7.9%)	70 (7.0%)	271 (8.1%)
Mental illness	758 (17.5%)	168 (16.9%)	590 (17.7%)
Depression	213 (4.9%)	50 (5.0%)	163 (4.9%)
Constipation	1626 (37.6%)	364 (36.7%)	1262 (37.9%)
Nausea/vomiting	399 (9.2%)	92 (9.3%)	307 (9.2%)
Diarrhea	89 (2.1%)	16 (1.6%)	73 (2.2%)
Treatments used at the index date			
RAASi	2983 (69.0%)	685 (69.0%)	2298 (69.1%)
ACEI	737 (17.1%)	167 (16.8%)	570 (17.1%)
ARB	2388 (55.3%)	552 (55.6%)	1836 (55.2%)
MRA	944 (21.8%)	186 (18.7%)	758 (22.8%)
SPS/CPS	4321 (100.0%)	993 (100.0%)	3328 (100.0%)
Laxative	2204 (51.0%)	487 (49.0%)	1717 (51.6%)
Antidiarrheal	821 (19.0%)	199 (20.0%)	622 (18.7%)
Antiemetic	949 (22.0%)	228 (23.0%)	721 (21.7%)
Phosphate binder	147 (3.4%)	33 (3.3%)	114 (3.4%)

Values are mean ± standard deviation or *n* (%).

^a^ Calculated using the number of patients with CKD, in each group as the denominator.

ACEI, angiotensin-converting enzyme inhibitor; ADL, activities of daily living score; ARB, angiotensin receptor blocker; CKD, chronic kidney disease; CPS, calcium polystyrene sulfonate; eGFR, estimated glomerular filtration rate; MPR, medication possession ratio; MRA, mineralocorticoid receptor antagonist; RAASi, renin–angiotensin–aldosterone system inhibitor; S-K, serum potassium; SPS, sodium polystyrene sulfonate.

### 2.3 Data analyses

Clinical outcome events of ER visits, hospitalization for any reason, HF, cardiac events, introduction of RRT, recurrence of HK, and eGFR decline were evaluated between the two groups. The time dependence of each group was tested using the log-rank test. Moreover, Cox proportional hazards models adjusted for baseline characteristics were conducted to evaluate adjusted hazard ratio (aHR) for the outcome events with reference to MPR ≥80% group. The adjusted Cox proportional models included following variables, such as age, sex, RAASi treatment, comorbidities (CKD, diabetes mellitus (DM), hypertension), Charlson comorbidity score, and use of loop and thiazide diuretics. For S-K levels, we calculated the least-squares mean of the test values at 3 ± 1M, 6 ± 1M, and 12 ± 1M and its 95% CI from the index date.

## 3 Results

### 3.1 Patient characteristics

The baseline characteristics of the study population are summarized in Table 1. The mean age of the overall population was 74.6 ± 12.83 years, and 57.3% were male. The mean eGFR was 28.5 ± 20.8 mL/min/1.73 m^2^. The most common comorbidities were hypertension (80.9%), CKD (69.8%; stage 4, 37.8%; stage 5, 37.6%), DM (56.6%), and HF (44.1%). Approximately two-thirds of patients (69.0%) were prescribed an RAASi at the index date, with angiotensin receptor blocker (ARB) being the most common prescription (55.3%). Table 1 also shows the characteristics of patients subdivided into MPR <80% (*n* = 993) and MPR ≥80% (*n* = 3,328) groups. Change in the number of treatments prescribed during the follow-up period are described in Table 2. As presented in these table, there were no obvious differences in the baseline characteristics, recorded diagnoses, or treatments prescribed between the two groups.

**TABLE 2:** Treatments prescribed during the follow-up period (overall population).

	Overall population MPR <80% (N = 993, 23.0%^a^)				Overall population MPR ≥80% (N = 3328, 77.0%^a^)			
	Index date	3M±1M	6M±1M	12M±1M	Index date	3M±1M	6M±1M	12M±1M
Chronic heart failure (HF)^b^								
Yes	419 (42.2)	337 (35.9)	332 (38.9)	270 (37.2)	1488 (44.7)	979 (35.3)	908 (35.7)	812 (36.2)
RAASi treatment ^b^,^c^	338 (34.0)	212 (22.6)	208 (24.4)	168 (23.1)	1221 (36.7)	651 (23.5)	576 (22.6)	510 (22.7)
ACE	101 (10.2)	58 (6.2)	54 (6.3)	44 (6.1)	390 (11.7)	166 (6.0)	141 (5.5)	125 (5.6)
ARB	247 (24.9)	147 (15.7)	146 (17.1)	118 (16.3)	873 (26.2)	450 (16.2)	422 (16.6)	370 (16.5)
MRA	144 (14.5)	62 (6.6)	44 (5.2)	30 (4.1)	620 (18.6)	166 (6.0)	137 (5.4)	115 (5.1)
Non-RAASi treatment for HF ^b^,^c^	384 (38.7)	276 (29.4)	263 (30.8)	218 (30.0)	1385 (41.6)	820 (29.6)	733 (28.8)	656 (29.2)
No	574 (57.8)	602 (64.1)	521 (61.1)	456 (62.8)	1840 (55.3)	1794 (64.7)	1638 (64.3)	1432 (63.8)
Hypertension								
Yes	810 (81.6)	682 (72.6)	634 (74.3)	526 (72.5)	2686 (80.7)	2038 (73.5)	1851 (72.7)	1603 (71.4)
No	183 (18.4)	257 (27.4)	219 (25.7)	200 (27.5)	642 (19.3)	735 (26.5)	695 (27.3)	641 (28.6)
Number of antihypertensive type (n, %)	791 (79.7)	636 (67.7)	567 (66.5)	474 (65.3)	2678 (80.5)	1909 (68.8)	1652 (64.9)	1393 (62.1)
Drugs inducing Hyper K (n, %)^b^								
RAASi treatment	685 (69.0)	483 (51.4)	444 (52.1)	354 (48.8)	2298 (69.1)	1499 (54.1)	1311 (51.5)	1108 (49.4)
ACE	167 (16.8)	92 (9.8)	84 (9.8)	67 (9.2)	570 (17.1)	296 (10.7)	255 (10.0)	216 (9.6)
ARB	552 (55.6)	396 (42.2)	362 (42.4)	289 (39.8)	1836 (55.2)	1215 (43.8)	1089 (42.8)	910 (40.6)
MRA	186 (18.7)	83 (8.8)	59 (6.9)	38 (5.2)	758 (22.8)	224 (8.1)	179 (7.0)	147 (6.6)
Non-RAASi treatment	832 (83.8)	647 (68.9)	566 (66.4)	484 (66.7)	2859 (85.9)	1860 (67.1)	1614 (63.4)	1384 (61.7)
Treatment (n, %)^b^								
Laxative								
Yes	487 (49.0)	250 (26.6)	236 (27.7)	167 (23.0)	1717 (51.6)	674 (24.3)	526 (20.7)	456 (20.3)
No	506 (51.0)	689 (73.4)	617 (72.3)	559 (77.0)	1611 (48.4)	2099 (75.7)	2020 (79.3)	1788 (79.7)
Antidiarrheal								
Yes	199 (20.0)	87 (9.3)	68 (8.0)	54 (7.4)	622 (18.7)	223 (8.0)	168 (6.6)	136 (6.1)
No	794 (80.0)	852 (90.7)	785 (92.0)	672 (92.6)	2706 (81.3)	2550 (92.0)	2378 (93.4)	2108 (93.9)
Antiemetic								
Yes	228 (23.0)	71 (7.6)	68 (8.0)	48 (6.6)	721 (21.7)	175 (6.3)	127 (5.0)	106 (4.7)
No	765 (77.0)	868 (92.4)	785 (92.0)	678 (93.4)	2607 (78.3)	2598 (93.7)	2419 (95.0)	2138 (95.3)
Phosphate binder								
Yes	33 (3.3)	48 (5.1)	54 (6.3)	59 (8.1)	114 (3.4)	162 (5.8)	157 (6.2)	155 (6.9)
No	960 (96.7)	891 (94.9)	799 (93.7)	667 (91.9)	3214 (96.6)	2611 (94.2)	2389 (93.8)	2089 (93.1)

^a^ Calculated by using the number of the total of the overall population as denominator.

^b^ Calculated by using the number of each MPR subgroup as denominator.

^c^ Number in HF patients.

The focused subpopulation was also divided into MPR <80% and MPR ≥80% groups. Their baseline characteristics and treatments prescribed during the follow-up period are presented in Supplementary Table S2, S3 and were generally similar to those of the overall study population.

### 3.2 Time to discontinuation of potassium binders

**FIGURE 2:**
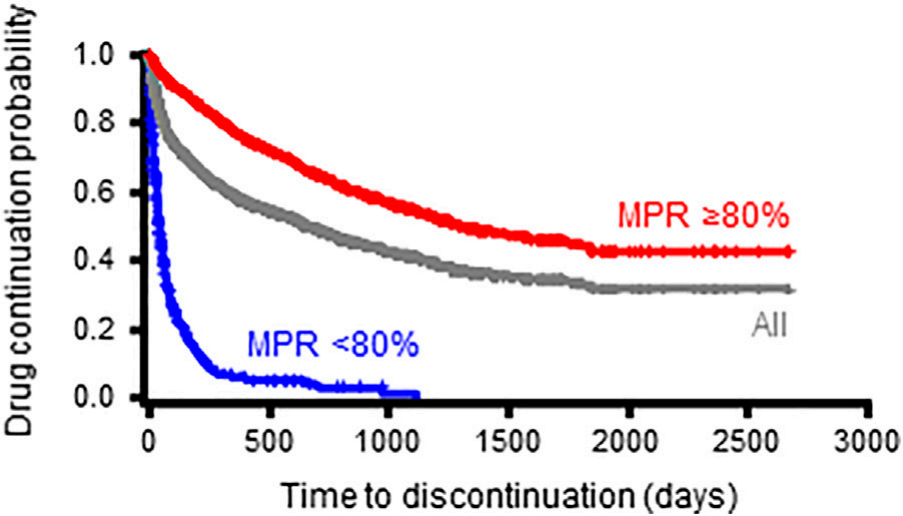
Time to discontinuation of PB therapy in the overall population (all patients and patients stratified by MPR).

The mean time to discontinuation of PBs from the index date was 881.6, 114.7, and 1,151.2 days in the overall population, MPR<80% group, and MPR ≥80% group, respectively (Table 3). In the MPR <80% group, the probability of discontinuation of PB increased sharply in the first 130 days, but in the MPR ≥80% group, the probability of discontinuation was moderate throughout the observation period, showing a gradient similar to the overall gradient (Figure 2).

**TABLE 3:** Time to discontinuation of a potassium binder in the overall population (all patients and patients stratified by MPR).

	Time to discontinuation, days		
	*N*	Mean (SD)	Median (95% CI^a^)
All	4321	881.6 (19.0)	655 (599–757)
MPR <80%	993	114.7 (9.1)	42 (36–47)
MPR ≥80%	3328	1151.2 (22.5)	1309 (1141–1731)

^a^ Wald 95% CI.

CI, confidence interval; MPR, medication possession ratio; SD, standard deviation.

The median time to discontinuation of PB therapy was 677 (95% CI 599–777) days in the focused subpopulation (Supplementary Table S4). Comparing median values for each MPR, the MPR <80% group had a significantly shorter time to discontinuation than the MPR ≥80% group (46 vs. 1,288 days, Supplementary Figure S2).

### 3.3 Serum potassium levels

**FIGURE 3:**
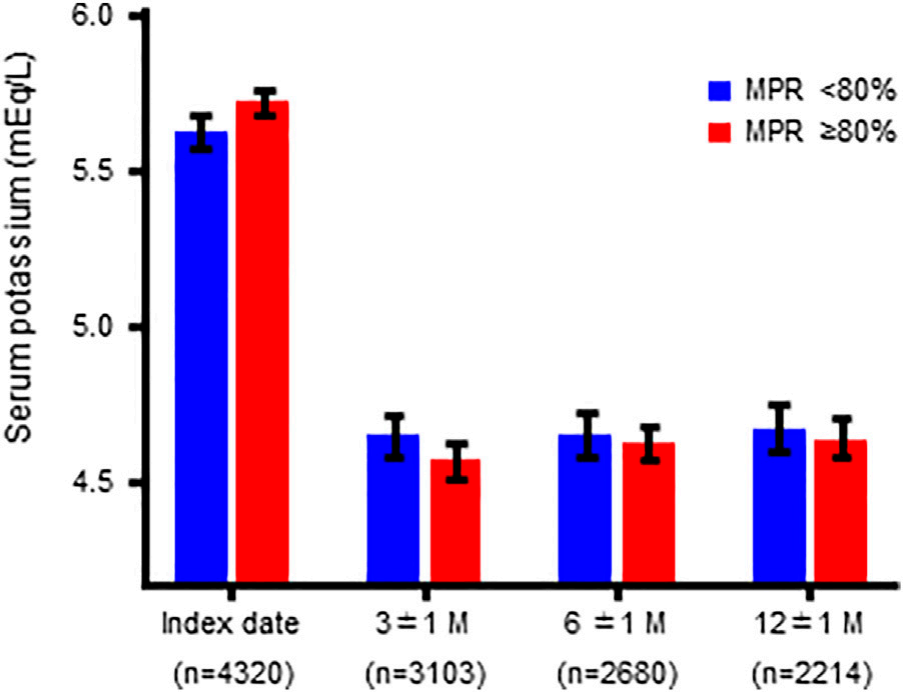
Serum potassium levels at the index date and at 3, 6, and 12 months in patients stratified by MPR (overall population).

The mean S-K level of the overall population at the index date was 5.71 ± 0.64 mEq/L. The S-K levels at the index date and at 3, 6, and 12 months in the MPR <80% and MPR ≥80% groups are shown in Figure 3 and Table 4.

**TABLE 4:** Serum potassium levels at the index date and at 3, 6, and 12 months in patients stratified by MPR (overall population).

	Serum potassium level, mEq/L				
	MPR <80%		MPR ≥80%		
	*N*	LS mean (95% CI)	*N*	LS mean (95% CI)	*p*-value
Index date	992	5.62 (5.57–5.68)	3328	5.72 (5.68–5.76)	<0.001
3 ± 1 M	788	4.65 (4.58–4.71)	2315	4.57 (4.51–4.62)	0.005
6 ± 1 M	700	4.65 (4.58–4.72)	1980	4.63 (4.57–4.68)	0.360
12 ± 1 M	567	4.67 (4.60–4.75)	1647	4.64 (4.58–4.70)	0.307

LS, means were adjusted for age, sex, renin–angiotensin–aldosterone system inhibitor use, comorbidities (chronic kidney disease, diabetes mellitus, heart failure, hypertension), loop diuretic use, and thiazide diuretic use.

CI, confidence interval; LS, least squares; M, months; MPR, medication possession ratio.

There were significant differences in the S-K levels at the index date and at 3 months, but not at 6 and 12 months, between the two groups. A similar trend was observed for the focused subpopulation (Supplementary Figure S3; Supplementary Table S5).

### 3.4 Clinical outcome in MPR≥80% and MPR<80% groups

**FIGURE 4:**
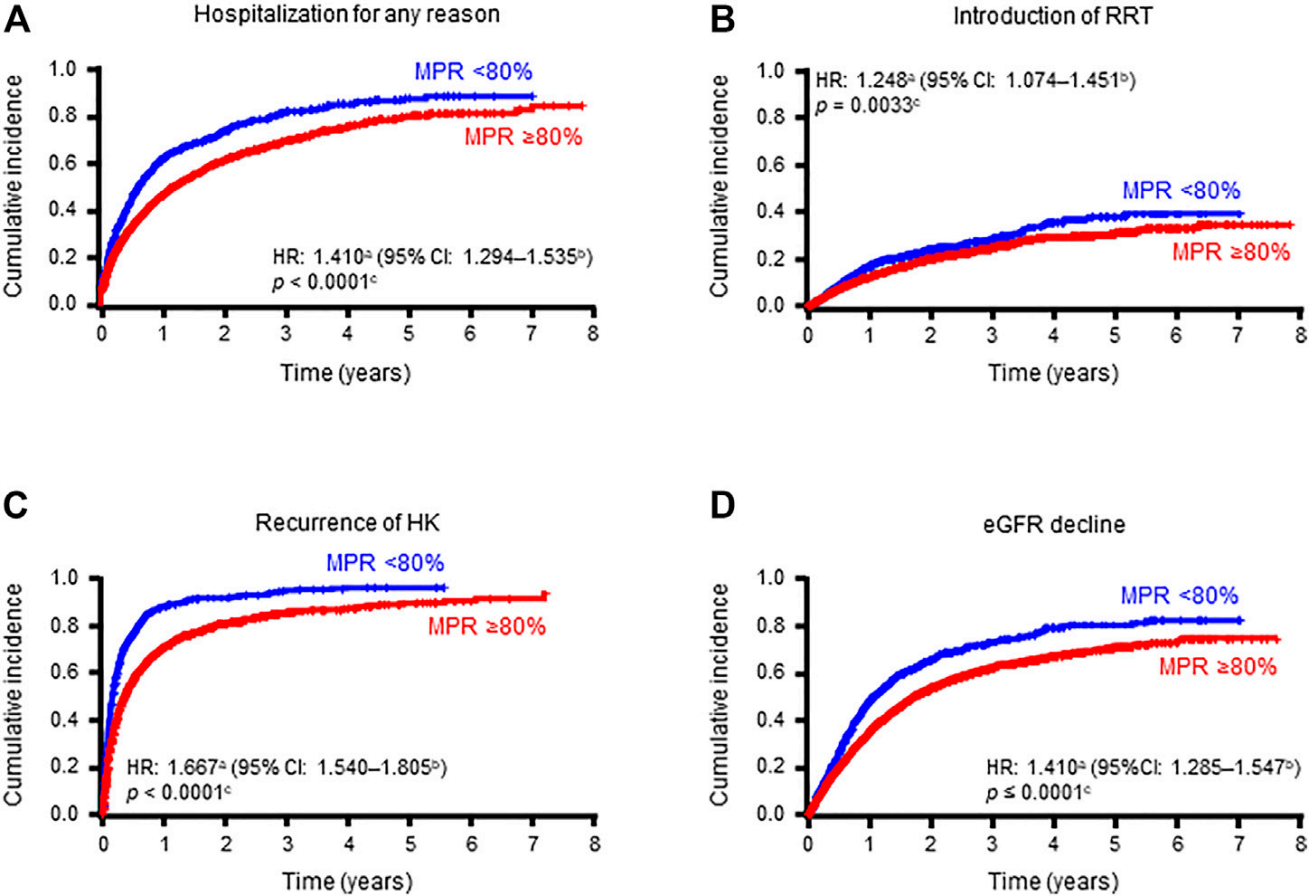
Cumulative incidences of hospitalization for any reason **(A)**, introduction of RRT **(B)**, recurrence of hyperkalemia **(C)**, and eGFR decline **(D)** in patients stratified by MPR (overall population).

The proportion of clinical events in the overall population and focused subpopulation was presented in the two groups: MPR≥80% and MPR<80% (Table 5)**.** In the overall population, significant increases in the event rate of ER visits, hospitalization for any reason, hospitalization for HF, hospitalization for cardiac events, introduction of RRT, CG-GI therapy, ICU admission, recurrence of HK, and eGFR decline were observed in the MPR<80% group compared to the MPR≥80% group, with hazard ratios (HRs) of 1.231, 1.410, 1.464, 1.363, 1.248, 1.228, 1.241, 1.667, and 1.410, respectively. In contrast, there were no significant differences in death or rehospitalization between the two groups. In the focused subpopulation, significant increases in hospitalization for any reason, induction of RRT, recurrent HK, and eGFR reduction were found reproducibly, with HRs of 1.208, 1.256, 1.452, and 1.300, respectively (Table 5; Figure 4).

**TABLE 5:** Association between the MPR for potassium binders and clinical events in patients with hyperkalemia (overall population and focused subpopulation).

Clinical event^a^	Overall population (*n* = 4321)				Focused subpopulation (*n* = 1945)^b^			
	MPR <80%	MPR ≥80%	HR^c^ (95% CI^d^)	*p* ^e^	MPR <80%	MPR ≥80%	HR^c^ (95% CI^d^)	*p* ^e^
*n* (%)	993 (23.0%)	3328 (77.0%)	—	—	475 (24.4%)	1470 (75.6%)	—	—
Death	160 (16.1%)	515 (15.5%)	1.002 (0.839–1.197)	0.983	43 (9.1%)	127 (8.6%)	1.176 (0.832–1.662)	0.359
ER visit	373 (37.6%)	1040 (31.3%)	1.231 (1.093–1.386)	<0.001	171 (36.0%)	552 (37.6%)	1.013 (0.852–1.204)	0.877
Hospitalization for any reason	727 (73.2%)	1932 (58.1%)	1.410 (1.294–1.535)	<0.001	351 (73.9%)	1015 (69.0%)	1.208 (1.067–1.367)	0.002
Hospitalization for HF	43 (4.3%)	98 (2.9%)	1.464 (1.024–2.094)	0.033	16 (3.4%)	58 (3.9%)	0.895 (0.514–1.558)	0.694
Hospitalization for cardiac events	155 (15.6%)	378 (11.4%)	1.363 (1.130–1.643)	0.001	73 (15.4%)	219 (14.9%)	1.084 (0.831–1.413)	0.547
Rehospitalization	209 (21.0%)	455 (13.7%)	1.061 (0.903–1.247)	0.474	100 (21.1%)	241 (16.4%)	1.053 (0.837–1.325)	0.664
Introduction of RRT	232 (23.4%)	623 (18.7%)	1.248 (1.074–1.451)	0.003	133 (28.0%)	365 (24.8%)	1.256 (1.028–1.534)	0.024
CG-GI therapy	264 (26.6%)	723 (21.7%)	1.228 (1.070–1.411)	0.003	106 (22.3%)	285 (19.4%)	1.227 (0.982–1.532)	0.071
ICU admission	163 (16.4%)	437 (13.1%)	1.241 (1.038–1.484)	0.017	65 (13.7%)	186 (12.7%)	1.153 (0.870–1.530)	0.319
Recurrence of hyperkalemia	867 (87.3%)	2277 (68.4%)	1.667 (1.540–1.805)	<0.001	442 (93.1%)	1298 (88.3%)	1.452 (1.298–1.626)	<0.001
eGFR decline	623 (62.7%)	1603 (48.2%)	1.410 (1.285–1.547)	<0.001	334 (70.3%)	924 (62.9%)	1.300 (1.143–1.479)	<0.001

^a^ See Supplementary Table S1 for definitions of clinical events.

^b^ Patients with follow-up data at all three time points (3, 6, and 12 months).

^c^ Cox proportional hazards ratio (reference group: MPR ≥80%) Death: Cox proportional hazards regression model. Non-death: Cumulative survival function (reference group: MPR ≥80%).

^d^ Wald 95% CI.

^e^ Graytest (log-rank test for death).

CG-GI, calcium gluconate or glucose plus insulin injection; CI, confidence interval; eGFR, estimated glomerular filtration rate; ER, emergency room; HF, heart failure; HR, hazard ratio; ICU, intensive care unit; MPR, medication possession ratio; RRT, renal replacement therapy; SD, standard deviation.

## 4 Discussion

In this study, we sought to understand the outcomes of long-term PB therapy in real-world clinical practice in Japan using MPR as an indicator of the prescription continuation rate. While few studies have examined the impact of chronic treatment with PB in actual clinical settings (Kovesdy et al., 2019; Kovesdy et al., 2020), many patients require long-term management of HK, including those with chronic and progressive diseases, such as HF and CKD. Although no causal relationship has been demonstrated, abnormal S-K levels are associated with increased mortality (Korgaonkar et al., 2010; Nakhoul et al., 2015; Luo et al., 2016; Collins et al., 2017; Krogager et al., 2017; Furuland et al., 2018; Kashihara et al., 2019). Therefore, the chronic management of HK may play an important role in potentially fatal outcomes. To compare patients with chronic PB treatment with those without, we divided patients into two groups based on MPR, using a threshold of 80% as an indicator of long-term and chronic use. A threshold of 80% is commonly used to ensure good adherence to medication (Karve et al., 2009; Baumgartner et al., 2018; Hartman et al., 2019). As prescriptions are not reusable for a refill in Japan, the MPR can be substituted as an indicator of the prescription continuation rate in real-world clinical practice (Kuwabara et al., 2015; Burge et al., 2016; Higa et al., 2022; Komatsu et al., 2022).

By using the threshold of MPR 80%, there was a significant difference in the median prescription days of PBs between the MPR<80% and MPR ≥80% groups (655 and 1,309 days, respectively). The MPR ≥80% group had higher baseline S-K levels (mean S-K 5.72mEq/L) than the MPR <80% group (mean S-K 5.62mEq/L). This difference was canceled after 12 months of follow-up (Figure 3; Table 4). It has also been observed that the decrease in S-K levels occurred in the first 3 months and then remained at a similar level at 6 and 12 months. Considering the possibility of additional bias related to incomplete follow-up with missing data, we repeated the analyses using the focused subpopulation, comprising 1,945 patients with follow-up data at all three time points and found similar results (Supplementary Figure S3; Supplementary Table S5). Given that the mean and median times to PB discontinuation in MPR <80% group were 114.7 and 42 days, respectively (Table 3), the stable S-K control after 3 months (about 90 days) suggested a possibility that some doctors stopped PBs prescription based on a determination that there had been a clinically meaningful response (e.g., return to normal). The fact that PB therapy was discontinued sooner in the MPR <80% group than in the MPR ≥80% group (Figure 2) also supported this hypothesis. Although the goal of lowering S-K to a certain level is a common practice for PB treatment, there seem to be two major management methods: one is to discontinue PB prescription when S-K drops to a target level, and the other is to continue the prescription to maintain S-K at the target level.

Next, we compared the incidence of clinically relevant events between the MPR ≥80% and MPR <80% groups. Despite numerically similar S-K levels between the two groups after 12 months of follow-up, the MPR <80% group had significantly increased event rates in HK recurrence, hospitalization for any reason, introduction of RRT, and eGFR decline compared to the MPR ≥80% group in both the overall population and focused subpopulation (Table 5; Figure 4).

Recurrence of HK is relatively common and affects clinical outcomes in patients with HK. In our previous study, the cumulative incidence of recurrent HK after 1 year was 55.6%, 19.9%, and 4.9% among patients with S-K levels of ≥5.1, ≥5.5, or ≥6.0 mEq/L, respectively (Kashihara et al., 2019). In another study, recurrence of HK was reported in up to 49% of patients within 6 months in a Danish study (Adelborg et al., 2019) and 35% of patients within 1 year in a Canadian study (Hundemer et al., 2021). These reports and our findings suggest that chronic PB treatment may be beneficial for the prevention of recurrent HK events compared with short-term PB treatment.

In addition to recurrent HK events, we observed an increased event rate of hospitalization in the MPR <80% group. Although we did not examine the cause of each hospitalization, it is presumed, based on our previous report, that severe events related to CKD, cardiac events including HF, and recurrent HK may be associated with hospitalization (Kanda et al., 2020). Indeed, a previous study demonstrated that patients with HK were more likely to have frequent hospitalizations or rehospitalizations and longer hospital stays than normokalemic patients (Kanda et al., 2020). The study also showed that CKD, HF, and number of recurrent HK episodes were the three most important factors associated with increased healthcare costs, including hospitalization costs. As the continuation of PB therapy reduced hospitalizations in the current study, chronic PB treatment may also contribute to the reduction of healthcare expenditure and resource utilization by decreasing HK-related events requiring hospitalization.

Chronic PB treatment was also associated with renal protection, as shown by a reduction in the introduction of RRT and eGFR decline. In our recent study of Japanese patients with CKD, HK was associated with a greater decline in renal function. In fact, eGFR change over 3 years in CKD patients with or without HK was −5.75 mL/min/1.73 m^2^ or −1.79 mL/min/1.73 m^2^, respectively (Kohsaka et al., 2021). Taken together with the current findings of renal protective effects with chronic PB treatment, long-term management of HK with chronic PB treatment could be considered, especially for patients with CKD. It is important to note that an increase in S-K levels is directly related to impaired potassium excretion due to worsening kidney function (Gilligan and Raphael, 2017), and worsening renal function can contribute to an increase in recurrent HK. Therefore, renal protection observed in the MPR ≥80% group compared to the MPR <80% group might also have effects on the reduction of recurrent HK and subsequent hospitalization. In addition, it may be possible that patients with high MPR may suggest proper medication follow up with regular hospital visit, and these patients may have better clinical outcomes related to better adherence of other medication. However, there were no obvious differences in the number of treatments prescribed during the follow-up period (Table 2; Supplementary Table S3).

Current guidelines highlight the role of PBs in managing HK and preventing its recurrence (McDonagh et al., 2021; Heidenreich et al., 2022). Newer PBs may also permit the continuation or intensification of RAASi therapy (Agarwal et al., 2021; Imamura et al., 2021; Montagnani et al., 2021; Williams et al., 2021; Esteban-Fernández et al., 2022), which will improve the outcome of HF patients, as suggested by the ESC guidelines (McDonagh et al., 2021). However, at the time of this study, the PBs available in Japan were SPS and CPS. Further studies including the newer-generation PB, SZC (Lokelma^®^, AstraZeneca), may show a greater difference in clinical outcomes by maintaining, initiating, or up-titrating RAASi (Linder et al., 2016; Imamura et al., 2021; Rastogi et al., 2021; Swanson et al., 2021; Williams et al., 2021).

Overall, these findings suggest that long-term PB therapy may be beneficial for reducing the incidence of clinically relevant events along with other medication for renal and cardiac protection, such as RAASi, and may have the potential to improve HK management for better prognosis in addition to ongoing S-K control.

## 5 Limitations

Some limitations of this study are inherent to the analyses of a hospital claims database, including the presence of unmeasured confounding factors, incomplete data, and the possibility that diagnoses and/or prescriptions may be inaccurate or vary by institution. Thus, measurements of variables may be incomplete and non-precise. Furthermore, patients were not tracked across multiple hospitals, and not all hospitals participated in the database. Thus, patients may receive prescriptions for PB at one hospital but may be admitted to another hospital for other reasons, such as treatment of HF or renal disease. Additionally, changes in concomitant medications, including RAASis or other drugs associated with an increased risk of HK, during the follow-up were not analyzed in this study. Likewise, the use of other S-K-lowering therapies has not been assessed. Finally, we have not considered the adverse effects of PBs in this study and since we used MPR for prescriptions length, it does not necessarily mean that the received medication was taken by the patients.

## 6 Conclusion

In conclusion, few studies have examined the impact of long-term treatment with PBs on clinical events in patients with HK in real-world clinical settings. Here, we have shown a reduction in the risk of clinically relevant events, including recurrent HK, hospitalization, introduction of RRT, and eGFR decline by chronic PB treatment with MPR ≥80% compared with MPR <80%. Chronic control with continued PBs rather than temporary treatment may be associated with the reduction of adverse clinical outcomes in patients with HK.

## Data Availability

The original contributions presented in the study are included in the article, further inquiries can be directed to the corresponding author.

## References

[B1] Adelborg K. Nicolaisen S. K. Hasvold P. Palaka E. Pedersen L. Thomsen R. W. (2019). Predictors for repeated hyperkalemia and potassium trajectories in high-risk patients - a population-based cohort study. PLoS One 14, e0218739. 10.1371/journal.pone.0218739 31226134 PMC6588240

[B2] Agarwal R. Rossignol P. Budden J. Mayo M. R. Arthur S. Williams B. (2021). Patiromer and spironolactone in resistant hypertension and advanced CKD: Analysis of the randomized AMBER trial. Kidney360. 2, 425–434. 10.34067/KID.0006782020 35369022 PMC8785994

[B3] Akaba Y. Nojo Y. Sakurai H. Masuyama K. (2019). Challenges of instituting a prescription refill system in Japan. Regul. Sci. Med. Prod. 9, 69–78.

[B4] Baumgartner P. C. Haynes R. B. Hersberger K. E. Arnet I. (2018). A systematic review of medication adherence thresholds dependent of clinical outcomes. Front. Pharmacol. 9, 1290. 10.3389/fphar.2018.01290 30524276 PMC6256123

[B5] Burge R. Sato M. Sugihara T. (2016). Real-world clinical and economic outcomes for daily teriparatide patients in Japan. J. Bone Min. Metab. 34, 692–702. 10.1007/s00774-015-0720-0 26661475

[B6] Clase C. M. Carrero J. J. Ellison D. H. Grams M. E. Hemmelgarn B. R. Jardine M. J. (2020). Potassium homeostasis and management of dyskalemia in kidney diseases: Conclusions from a kidney disease: Improving global outcomes (KDIGO) controversies conference. Kidney Int. 97, 42–61. 10.1016/j.kint.2019.09.018 31706619

[B7] Collins A. J. Pitt B. Reaven N. Funk S. McGaughey K. Wilson D. (2017). Association of serum potassium with all-cause mortality in patients with and without heart failure, chronic kidney disease, and/or diabetes. Am. J. Nephrol. 46, 213–221. 10.1159/000479802 28866674 PMC5637309

[B8] Desai N. R. Rowan C. G. Alvarez P. J. Fogli J. Toto R. D. (2020). Hyperkalemia treatment modalities: A descriptive observational study focused on medication and healthcare resource utilization. PLoS One 15, e0226844. 10.1371/journal.pone.0226844 31910208 PMC6946143

[B9] Esteban-Fernández A. Ortiz Cortés C. López-Fernández S. Recio Mayoral A. Camacho Jurado F. J. Gómez Otero I. (2022). Experience with the potassium binder patiromer in hyperkalaemia management in heart failure patients in real life. Esc. Heart Fail 9, 3071–3078. 10.1002/ehf2.13976 35748119 PMC9715760

[B10] Furuland H. McEwan P. Evans M. Linde C. Ayoubkhani D. Bakhai A. (2018). Serum potassium as a predictor of adverse clinical outcomes in patients with chronic kidney disease: New risk equations using the UK clinical practice research datalink. BMC Nephrol. 19, 211. 10.1186/s12882-018-1007-1 30134846 PMC6106824

[B11] Gilligan S. Raphael K. L. (2017). Hyperkalemia and hypokalemia in CKD: Prevalence, risk factors, and clinical outcomes. Adv. Chronic Kidney Dis. 24, 315–318. 10.1053/j.ackd.2017.06.004 29031358

[B12] Gorriz J. L. Muijsemberg A. Gimenez-Civera E. Perez A. Perez-Bernat E. Tomas P. (2019). “Characteristics of the population receiving treatment with calcium polystyrene sulfonate for hypercalemia. Low rate of compliance and collection of prescriptions in the pharmacy office,” in European renal association–European dialysis and transplant association (ERA–EDTA), 56th annual congress, 13–16 june 2019 (Budapest, Hungary.

[B13] Gumz M. L. Rabinowitz L. Wingo C. S. (2015). An integrated view of potassium homeostasis. N. Engl. J. Med. 373, 60–72. 10.1056/NEJMra1313341 26132942 PMC5675534

[B14] Hartman L. Lems W. F. Boers M. (2019). Outcome measures for adherence data from a medication event monitoring system: A literature review. J. Clin. Pharm. Ther. 44, 1–5. 10.1111/jcpt.12757 30171815 PMC7379515

[B15] Heidenreich P. A. Bozkurt B. Aguilar D. Allen L. A. Byun J. J. Colvin M. M. (2022). 2022 AHA/ACC/HFSA guideline for the management of heart failure: Executive summary: A report of the American College of Cardiology/American heart association joint committee on clinical practice guidelines. J. Am. Coll. Cardiol. 79, 1757–1780. 10.1016/j.jacc.2021.12.011 35379504

[B16] Higa S. Nakamura T. Ohwaki K. (2022). Inverse association between persistence with antidepressant medication and onset of chronic pain in patients with depression: A retrospective cohort study. J. Clin. Psychopharmacol. 42, 270–279. 10.1097/JCP.0000000000001544 35489030

[B17] Hundemer G. L. Talarico R. Tangri N. Leon S. J. Bota S. E. Rhodes E. (2021). Ambulatory treatments for RAAS inhibitor-related hyperkalemia and the 1-year risk of recurrence. Clin. J. Am. Soc. Nephrol. 16, 365–373. 10.2215/CJN.12990820 33608262 PMC8011018

[B18] Imamura T. Oshima A. Narang N. Kinugawa K. (2021). Clinical implications of sodium zirconium cyclosilicate therapy in patients with systolic heart failure and hyperkalemia. J. Clin. Med. 10, 5523. 10.3390/jcm10235523 34884224 PMC8658508

[B19] Japanese Society of Nephrology (2019). Essential points from evidence-based clinical practice guidelines for chronic kidney disease 2018. Clin. Exp. Nephrol. 23, 1–15. 10.1007/s10157-018-1648-1 30506489 PMC6344397

[B20] Kanda E. Kashihara N. Kohsaka S. Okami S. Yajima T. (2020). Clinical and economic burden of hyperkalemia: A nationwide hospital-based cohort study in Japan. Kidney Med. 2, 742–752.e1. 10.1016/j.xkme.2020.09.003 33319198 PMC7729225

[B21] Karve S. Cleves M. A. Helm M. Hudson T. J. West D. S. Martin B. C. (2009). Good and poor adherence: Optimal cut-point for adherence measures using administrative claims data. Curr. Med. Res. Opin. 25, 2303–2310. 10.1185/03007990903126833 19635045

[B22] Kashihara N. Kohsaka S. Kanda E. Okami S. Yajima T. (2019). Hyperkalemia in real-world patients under continuous medical care in Japan. Kidney Int. Rep. 4, 1248–1260. 10.1016/j.ekir.2019.05.018 31517144 PMC6734103

[B23] Kohsaka S. Okami S. Kanda E. Kashihara N. Yajima T. (2021). Cardiovascular and renal outcomes associated with hyperkalemia in chronic kidney disease: A hospital-based cohort study. Mayo Clin. Proc. Innov. Qual. Outcomes 5, 274–285. 10.1016/j.mayocpiqo.2020.10.001 33997627 PMC8105529

[B24] Komatsu Y. Yokoyama S. Hosomi K. Takada M. (2022). Impact of medication adherence on the association between oral anticoagulant use and risk of dementia: A retrospective cohort study using the Japanese claims database. Drugs Real World Outcomes 9, 437–449. 10.1007/s40801-022-00311-9 35717555 PMC9392663

[B25] Korgaonkar S. Tilea A. Gillespie B. W. Kiser M. Eisele G. Finkelstein F. (2010). Serum potassium and outcomes in CKD: Insights from the RRI-CKD cohort study. Clin. J. Am. Soc. Nephrol. 5, 762–769. 10.2215/CJN.05850809 20203167 PMC2863985

[B26] Kovesdy C. P. Gosmanova E. O. Woods S. D. Fogli J. J. Rowan C. G. Hansen J. L. (2020). Real-world management of hyperkalemia with patiromer among United States Veterans. Postgrad. Med. 132, 176–183. 10.1080/00325481.2019.1706920 31971043

[B27] Kovesdy C. P. Rowan C. G. Conrad A. Spiegel D. M. Fogli J. Oestreicher N. (2019). Real-world evaluation of patiromer for the treatment of hyperkalemia in hemodialysis patients. Kidney Int. Rep. 4, 301–309. 10.1016/j.ekir.2018.10.020 30775627 PMC6365398

[B28] Krogager M. L. Torp-Pedersen C. Mortensen R. N. Køber L. Gislason G. Søgaard P. (2017). Short-term mortality risk of serum potassium levels in hypertension: A retrospective analysis of nationwide registry data. Eur. Heart J. 38, 104–112. 10.1093/eurheartj/ehw129 28158516

[B29] Kuwabara H. Saito Y. Mahlich J. (2015). Adherence and rehospitalizations in patients with schizophrenia: Evidence from Japanese claims data. Neuropsychiatr. Dis. Treat. 11, 935–940. 10.2147/NDT.S81677 25897229 PMC4389915

[B30] Linder K. E. Krawczynski M. A. Laskey D. (2016). Sodium zirconium cyclosilicate (ZS-9): A novel agent for the treatment of hyperkalemia. Pharmacotherapy 36, 923–933. 10.1002/phar.1797 27393581

[B31] Luo J. Brunelli S. M. Jensen D. E. Yang A. (2016). Association between serum potassium and outcomes in patients with reduced kidney function. Clin. J. Am. Soc. Nephrol. 11, 90–100. 10.2215/CJN.01730215 26500246 PMC4702219

[B32] McDonagh T. A. Metra M. Adamo M. Gardner R. S. Baumbach A. Böhm M. (2021). 2021 ESC Guidelines for the diagnosis and treatment of acute and chronic heart failure. Eur. Heart J. 42, 3599–3726. 10.1093/eurheartj/ehab368 34447992

[B33] Montagnani A. Frasson S. Gussoni G. Manfellotto D. (2021). Optimization of RAASi therapy with new potassium binders for patients with heart failure and hyperkalemia: Rapid review and meta-analysis. J. Clin. Med. 10, 5483. 10.3390/jcm10235483 34884184 PMC8658658

[B34] Montford J. R. Linas S. (2017). How dangerous is hyperkalemia? J. Am. Soc. Nephrol. 28, 3155–3165. 10.1681/ASN.2016121344 28778861 PMC5661285

[B35] Mu F. Betts K. A. Woolley J. M. Dua A. Wang Y. Zhong J. (2020). Prevalence and economic burden of hyperkalemia in the United States Medicare population. Curr. Med. Res. Opin. 36, 1333–1341. 10.1080/03007995.2020.1775072 32459116

[B36] Nakhoul G. N. Huang H. Arrigain S. Jolly S. E. Schold J. D. Nally J. V. Jr. (2015). 'Serum potassium, end-stage renal disease and mortality in chronic kidney disease. Am. J. Nephrol. 41, 456–463. 10.1159/000437151 26228532 PMC4686260

[B37] Palmer B. F. Clegg D. J. (2016). Achieving the benefits of a high-potassium, paleolithic diet, without the toxicity. Mayo Clin. Proc. 91, 496–508. 10.1016/j.mayocp.2016.01.012 26948054

[B38] Rastogi A. Hanna R. M. Mkrttchyan A. Khalid M. Yaqoob S. Shaffer K. (2021). Sodium zirconium cyclosilicate for the management of chronic hyperkalemia in kidney disease, a novel agent. Expert Rev. Clin. Pharmacol. 14, 1055–1064. 10.1080/17512433.2021.1932460 34227913

[B39] Ren H. Leon S. J. Whitlock R. Rigatto C. Komenda P. Bohm C. (2022). Prescription patterns of sodium and calcium polystyrene sulfonate in patients with hyperkalemia and chronic kidney disease receiving RAAS inhibitors. Clin. Kidney J. 15, 1713–1719. 10.1093/ckj/sfac077 36003673 PMC9394712

[B40] Rossignol P. Ruilope L. M. Cupisti A. Ketteler M. Wheeler D. C. Pignot M. (2020). Recurrent hyperkalaemia management and use of renin-angiotensin-aldosterone system inhibitors: A European multi-national targeted chart review. Clin. Kidney J. 13, 714–719. 10.1093/ckj/sfz129 32905252 PMC7467623

[B41] Sarwar C. M. Papadimitriou L. Pitt B. Piña I. Zannad F. Anker S. D. (2016). Hyperkalemia in heart failure. J. Am. Coll. Cardiol. 68, 1575–1589. 10.1016/j.jacc.2016.06.060 27687200

[B42] Swanson K. J. Aziz F. Parajuli S. Mohamed M. Mandelbrot D. A. Djamali A. (2021). Sodium zirconium cyclosilicate use in kidney transplant recipients. Nephrol. Dial. Transpl. 36, 2151–2153. 10.1093/ndt/gfab172 33914876

[B43] Tanaka S. Seto K. Kawakami K. (2015). Pharmacoepidemiology in Japan: Medical databases and research achievements. J. Pharm. Health Care Sci. 1, 16. 10.1186/s40780-015-0016-5 26819727 PMC4729130

[B44] Williams R. James A. Ashton M. Vaughan S. Wong A. (2021). Use of sodium zirconium cyclosilicate for up-titration of renin-angiotensin-aldosterone system inhibitor therapy in patients with heart failure: A case series. Eur. Heart J. Case Rep. 5, ytab281. 10.1093/ehjcr/ytab281 34409249 PMC8364764

