# Supplementary material for: Impact of chronic potassium binder treatment on the clinical outcomes in patients with hyperkalemia: Results of a nationwide hospital-based cohort study
Source: Front Physiol. 2023 Apr 12;14:1156289. doi: 10.3389/fphys.2023.1156289 (PMC10130648; doi:10.3389/fphys.2023.1156289)
Supplement: Supplementary file 1 [file DataSheet1.docx]

**Impact of chronic potassium binder treatment on the clinical outcomes in patients with hyperkalemia: Results of a nationwide hospital-based cohort study**

**Eiichiro Kanda^1^, Naru Morita^2^, Toshitaka Yajima^2*^**

^1^Medical Science, Kawasaki Medical School, Kurashiki, Okayama, Japan

^2^Cardiovascular, Renal, and Metabolism, Medical Affairs, AstraZeneca K.K., Osaka, Japan

## *Correspondence:

Toshitaka Yajima

e-mail: Toshitaka.Yajima@astrazeneca.com

# Supplementary Material

**Figure S1.** Definitions of the index date, look-back period, and follow-up period


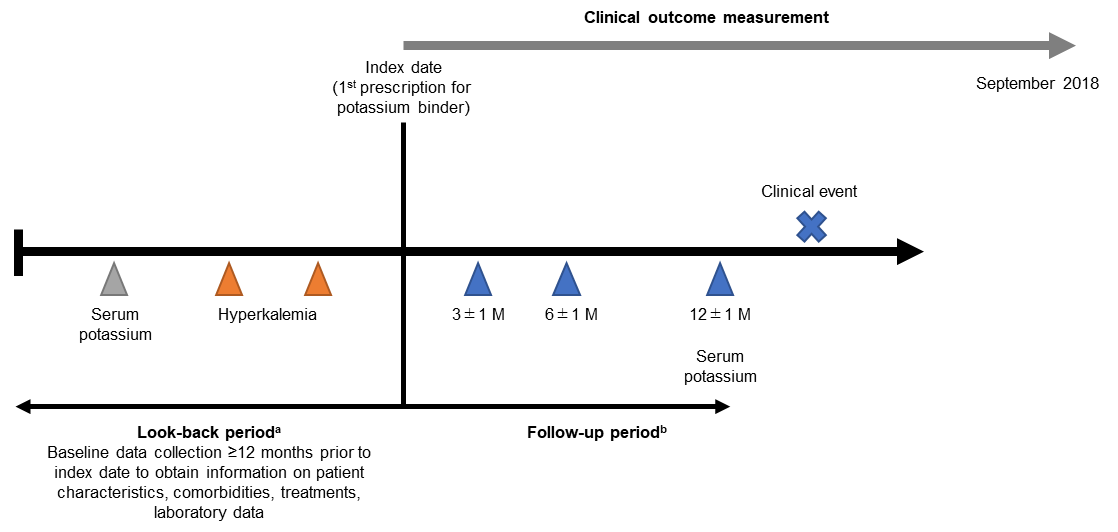


^a^Patients with hyperkalemia were defined as those with at least two serum potassium values of ≥5.1 mEq/L within 1 year (≤360 days). Hyperkalemia episodes may occur after the index date.

^b^Medication records in the follow-up period were used to calculate the medication possession ratio (MPR) as the proportion of the number of days the drug was prescribed to the duration of follow-up.

**Figure S2.** Time to discontinuation of potassium binder therapy in the focused data subpopulation (all patients and patients stratified by MPR)


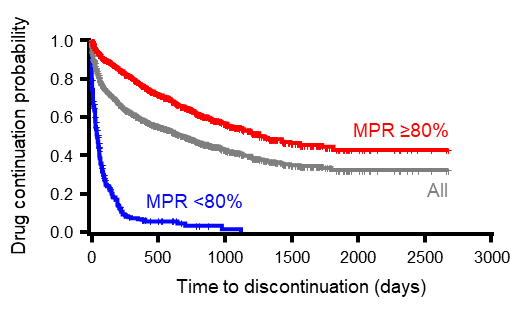


**Figure S3.** Serum potassium levels at the index date and at 3, 6, and 12 months in patients stratified by MPR (focused subpopulation)

**
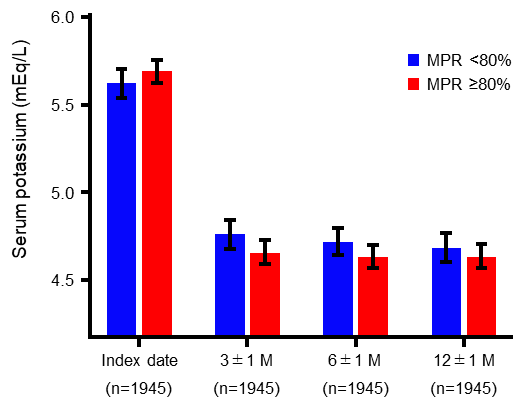
**

**Table S1.** Definition of clinical events

| **Event** | **Definition of event^a^** | **Date of event** |
| --- | --- | --- |
| Death | DPC hospitalization with an outcome of death. | Date of discharge |
| ER visit | Medical record with claim code A205 or A300 | Date of the medical action |
| Hospitalization for any reason | Medical record associated with hospitalization^b^ | Date of the medical action |
| Hospitalization for HF | Hospitalization associated with a diagnosis of HF (I50, I110)^b^ | Date of the medical action |
| Hospitalization for cardiac events | Hospitalization associated with a diagnosis of a cardiac event (I21, I22, I23, I44, I45, I46, I47, I48, I49)^b^ | Date of starting treatment for the event |
| Rehospitalization | Hospitalization for any reason within 30 days after discharge for prior hospitalization, for patients with outcome other than death. The time from discharge for first hospitalization to rehospitalization was recorded | Date of the medical action |
| Introduction of RRT | Claim code for dialysis or kidney transplantation | Date of the medical action |
| CG-GI therapy | Claim code for either of the following:   - Calcium gluconate (CG) - Glucose and insulin (GI) injection on the same day | Date of prescription |
| ICU admission | Medical record with claim code for ICU admission | Date of the medical action |
| Recurrence of hyperkalemia | S-K level ≥5.1 mEq/L recorded after a normal level (3.6–5.0 mEq/L). Analysis limited to patients with normal S-K level during the follow-up period. The time from the date of measurement of the first normal S-K level to the elevated S-K level during the follow-up was recorded | Date of the value ≥5.1 mEq/L |
| eGFR decline | Decline in eGFR value by ≥40% within 2 years after the index date |  |

^a^National codes used in the DPC system

^b^Hospitalization prior to the index date was not considered as an event

eGFR, estimated glomerular filtration rate; ER, emergency room; HF, heart failure; ICU, intensive care unit; RRT, renal replacement therapy

**Table S2.** Patient characteristics at the index date (focused subpopulation)

|  | **All patients** | **MPR <80%** | **MPR ≥80%** |
| --- | --- | --- | --- |
|  | **(*n* = 1945)** | **(*n* = 475)** | **(*n* = 1470)** |
| Age (years) | 71.4 ± 12.23 | 71.1 ± 11.56 | 71.5 ± 12.45 |
| Sex, male | 1196 (61.5%) | 298 (62.7%) | 898 (61.1%) |
| ADL | 16.5 ± 6.19 | 16.5 ± 6.15 | 16.5 ± 6.2 |
| eGFR (mL/min/1.73 m^2^) | 26.9 ± 17.8 | 26.5 ± 18.1 | 27.0 ± 17.6 |
| Recorded diagnoses |  |  |  |
| Diabetes mellitus | 1247 (64.1%) | 312 (65.7%) | 935 (63.6%) |
| Hypertension | 1719 (88.4%) | 421 (88.6%) | 1298 (88.3%) |
| Heart failure | 798 (41.0%) | 182 (38.3%) | 616 (41.9%) |
| CKD | 1558 (80.1%) | 375 (78.9%) | 1183 (80.5%) |
| Stage 1^a^ | 7 (0.4%) | 2 (0.5%) | 5 (0.4%) |
| Stage 2^a^ | 39 (2.5%) | 5 (1.3%) | 34 (2.9%) |
| Stage 3a^a^ | 90 (5.8%) | 20 (5.3%) | 70 (5.9%) |
| Stage 3b^a^ | 262 (16.8%) | 57 (15.2%) | 205 (17.3%) |
| Stage 4^a^ | 638 (40.9%) | 161 (42.9%) | 477 (40.3%) |
| Stage 5^a^ | 522 (33.5%) | 130 (34.7%) | 392 (33.1%) |
| Myocardial infarction | 98 (5.0%) | 24 (5.1%) | 74 (5.0%) |
| Peripheral vascular disease | 476 (24.5%) | 113 (23.8%) | 363 (24.7%) |
| Cerebrovascular disease | 485 (24.9%) | 117 (24.6%) | 368 (25.0%) |
| Chronic pulmonary disease | 355 (18.3%) | 108 (22.7%) | 247 (16.8%) |
| Atrial fibrillation/atrial flutter | 258 (13.3%) | 59 (12.4%) | 199 (13.5%) |
| Obesity | 16 (0.8%) | 3 (0.6%) | 13 (0.9%) |
| Acute kidney injury | 129 (6.6%) | 34 (7.2%) | 95 (6.5%) |
| Mental illness | 273 (14.0%) | 65 (13.7%) | 208 (14.1%) |
| Depression | 71 (3.7%) | 19 (4.0%) | 52 (3.5%) |
| Constipation | 741 (38.1%) | 179 (37.7%) | 562 (38.2%) |
| Nausea/vomiting | 187 (9.6%) | 51 (10.7%) | 136 (9.3%) |
| Diarrhea | 41 (2.1%) | 6 (1.3%) | 35 (2.4%) |
| Treatments used at the index date |  |  |  |
| RAASi | 1462 (75.2%) | 361 (76.0%) | 1101 (74.9%) |
| ACEI | 348 (17.9%) | 85 (17.9%) | 263 (17.9%) |
| ARB | 1248 (64.2%) | 304 (64.0%) | 944 (64.2%) |
| MRA | 373 (19.2%) | 73 (15.4%) | 300 (20.4%) |
| SPS/CPS | 1945 (100.0%) | 475 (100.0%) | 1470 (100.0%) |
| Laxative | 902 (46.4%) | 219 (46.1%) | 683 (46.5%) |
| Antidiarrheal | 351 (18.0%) | 98 (20.6%) | 253 (17.2%) |
| Antiemetic | 399 (20.5%) | 107 (22.5%) | 292 (19.9%) |
| Phosphate binder | 73 (3.8%) | 18 (3.8%) | 55 (3.7%) |

Values are mean ± standard deviation or *n* (%)

^a^Calculated using the number of patients with CKD in each group as the denominator

ACEI, angiotensin converting enzyme inhibitor; ADL, Activities of Daily Living score; ARB, angiotensin receptor blocker; CKD, chronic kidney disease; CPS, calcium polystyrene sulphonate; eGFR, estimated glomerular filtration rate; MPR, medication possession ratio; MRA, mineralocorticoid receptor antagonist; RAASI, renin–angiotensin–aldosterone system inhibitor; S-K, serum potassium; SPS, sodium polystyrene sulphonate.

**Table S3.** Treatments prescribed during the follow-up period (Focused subpopulation)

|  | Focused subpopulation  MPR < 80% (N=475, 24.4% ^a^ ) | | | | Focused subpopulation  MPR ≥ 80% (N=1470, 75.6% ^a^ ) | | | |
| --- | --- | --- | --- | --- | --- | --- | --- | --- |
|  | Index date | 3M±1M | 6M±1M | 12M±1M | Index date | 3M±1M | 6M±1M | 12M±1M |
| Chronic heart failure (HF) |  |  |  |  |  |  |  |  |
| Yes | 182 (38.3) | 183 (38.5) | 193 (40.6) | 204 (42.9) | 616 (41.9) | 585 (39.8) | 616 (41.9) | 652 (44.4) |
| RAASi treatment ^b,c^ | 153 (32.2) | 128 (26.9) | 134 (28.2) | 133 (28.0) | 534 (36.3) | 418 (28.4) | 432 (29.4) | 440 (29.9) |
| ACE | 43 (9.1) | 34 (7.2) | 36 (7.6) | 34 (7.2) | 160 (10.9) | 106 (7.2) | 104 (7.1) | 108 (7.3) |
| ARB | 118 (24.8) | 91 (19.2) | 94 (19.8) | 94 (19.8) | 414 (28.2) | 298 (20.3) | 325 (22.1) | 323 (22.0) |
| MRA | 56 (11.8) | 31 (6.5) | 23 (4.8) | 21 (4.4) | 250 (17.0) | 106 (7.2) | 102 (6.9) | 97 (6.6) |
| Non-RAASi treatment for HF ^b,c^ | 169 (35.6) | 154 (32.4) | 158 (33.3) | 171 (36.0) | 579 (39.4) | 513 (34.9) | 529 (36.0) | 556 (37.8) |
| No | 293 (61.7) | 292 (61.5) | 282 (59.4) | 271 (57.1) | 854 (58.1) | 885 (60.2) | 854 (58.1) | 818 (55.6) |
| Hypertension |  |  |  |  |  |  |  |  |
| Yes | 421 (88.6) | 422 (88.8) | 425 (89.5) | 416 (87.6) | 1298 (88.3) | 1299 (88.4) | 1318 (89.7) | 1303 (88.6) |
| No | 54 (11.4) | 53 (11.2) | 50 (10.5) | 59 (12.4) | 172 (11.7) | 171 (11.6) | 152 (10.3) | 167 (11.4) |
| Number of antihypertensive type (n, %) | 405 (85.3) | 381 (80.2) | 386 (81.3) | 384 (80.8) | 1250 (85.0) | 1208 (82.2) | 1210 (82.3) | 1205 (82.0) |
| Drugs inducing Hyper K (n, %) ^b^ |  |  |  |  |  |  |  |  |
| RAASi treatment | 361 (76.0) | 297 (62.5) | 308 (64.8) | 289 (60.8) | 1101 (74.9) | 969 (65.9) | 959 (65.2) | 939 (63.9) |
| ACE | 85 (17.9) | 54 (11.4) | 59 (12.4) | 53 (11.2) | 263 (17.9) | 188 (12.8) | 182 (12.4) | 179 (12.2) |
| ARB | 304 (64.0) | 249 (52.4) | 255 (53.7) | 238 (50.1) | 944 (64.2) | 806 (54.8) | 812 (55.2) | 782 (53.2) |
| MRA | 73 (15.4) | 40 (8.4) | 30 (6.3) | 27 (5.7) | 300 (20.4) | 136 (9.3) | 127 (8.6) | 120 (8.2) |
| Non-RAASi treatment | 403 (84.8) | 358 (75.4) | 360 (75.8) | 372 (78.3) | 1247 (84.8) | 1126 (76.6) | 1125 (76.5) | 1139 (77.5) |
| Treatment (n, %) ^b^ |  |  |  |  |  |  |  |  |
| Laxative |  |  |  |  |  |  |  |  |
| Yes | 219 (46.1) | 110 (23.2) | 136 (28.6) | 106 (22.3) | 683 (46.5) | 352 (23.9) | 335 (22.8) | 361 (24.6) |
| No | 256 (53.9) | 365 (76.8) | 339 (71.4) | 369 (77.7) | 787 (53.5) | 1118 (76.1) | 1135 (77.2) | 1109 (75.4) |
| Antidiarrheal |  |  |  |  |  |  |  |  |
| Yes | 98 (20.6) | 34 (7.2) | 32 (6.7) | 44 (9.3) | 253 (17.2) | 114 (7.8) | 111 (7.6) | 111 (7.6) |
| No | 377 (79.4) | 441 (92.8) | 443 (93.3) | 431 (90.7) | 1217 (82.8) | 1356 (92.2) | 1359 (92.4) | 1359 (92.4) |
| Antiemetic |  |  |  |  |  |  |  |  |
| Yes | 107 (22.5) | 29 (6.1) | 31 (6.5) | 36 (7.6) | 292 (19.9) | 71 (4.8) | 76 (5.2) | 83 (5.6) |
| No | 368 (77.5) | 446 (93.9) | 444 (93.5) | 439 (92.4) | 1178 (80.1) | 1399 (95.2) | 1394 (94.8) | 1387 (94.4) |
| Phosphate binder |  |  |  |  |  |  |  |  |
| Yes | 18 (3.8) | 23 (4.8) | 28 (5.9) | 49 (10.3) | 55 (3.7) | 83 (5.6) | 103 (7.0) | 147 (10.0) |
| No | 457 (96.2) | 452 (95.2) | 447 (94.1) | 426 (89.7) | 1415 (96.3) | 1387 (94.4) | 1367 (93.0) | 1323 (90.0) |

a: Calculated by using the number of the total of the overall population as denominator.

b: Calculated by using the number of each MPR subgroup as denominator.

c: Number in HF patients.

**Table S4.** Time to discontinuation of potassium binder therapy in the focused subpopulation (all patients and patients stratified by MPR)

|  | Time to discontinuation, days | | |
| --- | --- | --- | --- |
|  | *n* | Mean (SD) | Median (95% CI ^a^) |
| All | 1945 | 870.5 (21.4) | 677 (599–777) |
| MPR <80% | 475 | 116.0 (10.6) | 46 (36–56) |
| MPR ≥80% | 1470 | 1125.4 (24.4) | 1288 (1122–1731) |

^a^Wald 95% CI

CI, confidence interval; MPR, medication possession ratio; SD, standard deviation

**Table S5.** Serum potassium levels at the index date and at 3, 6, and 12 months in patients stratified by MPR (focused subpopulation)

|  | Serum potassium level, mEq/L | | | |  |
| --- | --- | --- | --- | --- | --- |
|  |  | | | |  |
|  | MPR <80% | | MPR ≥80% | |  |
|  | *n* | LS mean (95% CI) | *n* | LS mean (95% CI) | *p*-value |
| Index date | 475 | 5.62 (5.54–5.70) | 1470 | 5.69 (5.62–5.76) | 0.036 |
| 3±1 M | 475 | 4.76 (4.68–4.84) | 1470 | 4.66 (4.59–4.73) | 0.003 |
| 6±1 M | 475 | 4.72 (4.64–4.80) | 1470 | 4.63 (4.57–4.70) | 0.009 |
| 12±1 M | 475 | 4.68 (4.60–4.77) | 1470 | 4.63 (4.57–4.70) | 0.143 |

LS means were adjusted for age, sex, renin–angiotensin–aldosterone system inhibitor use, comorbidities (chronic kidney disease, diabetes mellitus, heart failure, hypertension), loop diuretic use, and thiazide diuretic use.

CI, confidence interval; LS, least squares; M, months; MPR, medication possession ratio
